# Using Olfaction and Unpleasant Reminders to Reduce the Intention-behavior Gap in Hand Washing

**DOI:** 10.1038/srep18890

**Published:** 2016-01-06

**Authors:** Robert Pellegrino, Philip G. Crandall, Han-Seok Seo

**Affiliations:** 1 Department of Food Science, University of Arkansas, Fayetteville, AR 72704.

## Abstract

Lack of hand washing is a leading cause of food borne illnesses. To successfully increase hand hygiene compliance, interventions must have continual engagement with employees. This study used a real-time prospective memory (PM) scenario to measure the effectiveness of a control and sensory reminders of disgust to influence hand washing behavior and performance. First, a model of hand washing performance was built by having six participants’ hands contaminated with GermGlo (a florescent micro-particle) and then washed their hands using predetermined protocols while monitored by an electronic hand hygiene verification (HHV) system. Next, eighty Hispanic/Latino participants, in a between-group experimental design, performed a PM experiment while one of four reminders were present (hand washing poster, disgusting image, disgusting sound, and disgusting odor) as the HHV recorded their hand washing performance. Visual cues, typical of hand washing campaigns, were not as effective at increasing hand hygiene compliance as disgust-induced sensory cues. Furthermore, olfactory disgust showed a significantly higher probability that individuals would engage in hand washing behaviors than all other conditions. This study provides new insight into the effectiveness of different senses and emotion to reduce the intention-behavior gap associated with modifying behaviors, and broadens current PM research to a real-time application.

Food borne diseases are a serious public health concern in the United States and worldwide^1^,^2^. So what can be done to minimize the risk of consumers becoming ill when they go out to eat or buy fresh produce? Numerous risk assessments point to proper hand hygiene as an effective way of minimizing the risk from food borne pathogens in the food supply chain and reducing the danger of foods prepared in the food service industry^3^,^4^. Consequently, employers all along the food chain from farms, food processing facilities to wholesale and retail food service establishments have focused on increasing employees’ compliance with proper hand hygiene^5^,^6^.

Behavior models provide a framework for improved employee motivation, training, and education. Their uses have led to a considerable improvement in proper hand hygiene compliance. Popular behavior models based on the Theory of Planned Behavior (TPB)^7^, Organizational Theory^8^, and Health Behavior Model (HBM)^9^ have shown increased employee compliance. A meta-analysis of food safety training on hand hygiene knowledge and attitudes among food handlers found that a well-planned combination of both standard training and behavioral interventions were the most effective at improving hand hygiene engagement^10^. For instance, out of the five studies measuring hand hygiene attitudes, social cognitive intervention based on TPB and HBM, in combination with training, resulted in the highest shift of increased attitude toward hand hygiene.

According the Food and Drug Administration (FDA)^11^, increased hand washing performance in parallel with compliance should be the driving force in hand washing interventions to minimize the risk of foodborne disease. However, seldom do intervention studies reported in the literature measure the effectiveness of increasing hand washing performance (e.g., “*How well did they wash their hands?* ”) rather than just reporting an observation that they did wash their hands. For example, Green *et al.* (2006)^12^ monitored hand washing practices in 300 restaurants across six states and reported that only 1/3 of employees washed their hands after food contact activities that would have required hand washing, and of those attempts, only 27% of the time did employees wash their hands properly. Additionally, York *et al.* (2009)^13^ pointed out that long-term success in something as routine as hand washing requires multiple interventions in which hand washing components are incorporated into the environment, displaying posters and reminders (in culturally appropriate ways including making the reminders in the workers’ native language). These reminders reinforce basic concepts and emphasize desired behavior thus reducing the intention-behavior gap encountered during behavioral change, and help lead an employee to the ultimate goal of having the habit of proper hand hygiene^14^,^15^.

Typical hand washing reminders used during or after training have concentrated on two senses, sight and sound, both of which have been shown to increase proper hand hygiene behaviors. Early reminders were fashioned as educational tools; however, recent research shows more sophisticated reminders that are based on behavioral theories and targeting the employees’ emotions can be more effective^16^,^17^,^18^,^19^. For instance, in a hospital environment McGuckin *et al.* (2006) used role-modeling by using voice prompts recorded by different authoritative figures (e.g., shift managers) as hand hygiene reminders. Through this approach, the hospital saw a 60% overall increase in hand soap and sanitizer usage. Similarly, roles of disgust have been used to influence individuals through visual prompts^17^,^19^. Judah *et al.* (2009) placed electronic screens above the entry of several highway service station restrooms (collecting 200,000 restroom uses) and demonstrated that disgusting text prompts (e.g., “*Don’t take the loo with you—wash with soap*”) compared to the control condition, significantly increased soap usage by 9.8% for men. Additionally, Pellegrino *et al.* (2015)^20^ showed that individuals, both Caucasian and Hispanic, handling less hazardous food (fresh produce) were more likely to wash their hands as their feeling of disgust increased. However, there is limited research showing that disgust as a motivator can be leveraged to increase hand hygiene behaviors through simple reminders. Meanwhile, there has been recent research focusing on the potential of smell to influence behaviors related to self-protection^21^,^22^,^23^. Unlike other senses of sight and sound (which are limited by wavelengths and oscillation of air pressure), the hundreds of smell receptors may discriminate at least 1 trillion olfactory stimuli^24^, has long been tied to memory association^25^,^26^,^27^, and represents a first-line of defense for encountering danger^23^,^28^. For example, Olsson *et al.* (2014) injected healthy individuals with an endotoxin (activating the innate immune system) and a placebo to measure differences in behavioral immune response. They were able to demonstrate early chemosensory detection of smell among healthy individuals who rated odors of endotoxin-exposed individuals as more unpleasant, intense and unhealthy thus providing an avoidance mechanism for sickness.

This current study was designed to determine whether participants’ hand washing behavior is affected by sensory cue-based prospective memory (PM). More specifically, in the first part of the study (Experiment 1), a statistical model of hand washing performance was built using electronic hand hygiene verification (HHV) machine and GermGlo (a florescent micro-particle). Here, six participants’ hands were contaminated with GermGlo as a model system and asked to wash their hands for different amounts of time (5, 10, and 15 seconds) and residual amount of GermGlo was monitored by HHV. In Experiment 2, 80 Hispanic / Latino participants, the second largest ethnic group in the United States food workforce, performed a real-time PM task under four different treatment conditions (visual control, visual disgust, auditory disgust, and olfactory disgust) while the HHV recorded their hand washing performance. We hypothesize that disgust-induced reminders would be more effective at increasing hygiene behaviors than typical visual reminders, with the smell of disgust being the most effective due to its association with self-protection.

## Results

### Prospective Memory Sensory Reminders

**Figure 1:**
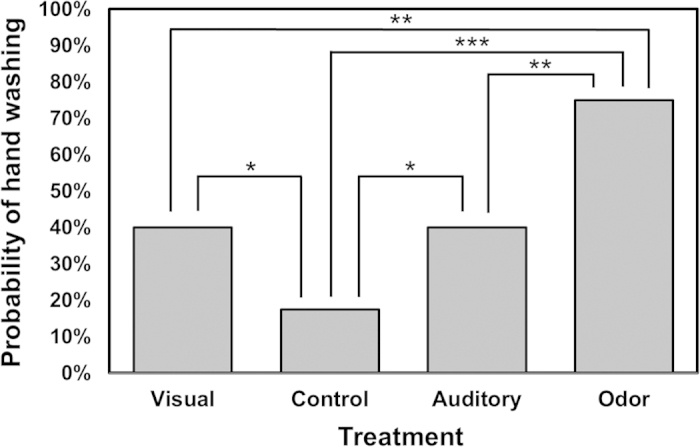
Logistic regression analysis showed the probability for individuals to wash their hands was different across the four conditions: control, visual, auditory, and odor (*p* < 0.001). Compared to the control, visual and auditory disgust conditions showed a significant increase in Prospective Memory (PM) task initiation (*p* < 0.05 each) while odor showed a larger significant increase (*p* < 0.001). Additionally, odor was significantly larger than both the visual and auditory disgust conditions (*p* < 0.01).

The probability an individual washed their hands significantly differed among the four reminder conditions used in the prospective memory scenario (control, visual disgust, auditory disgust, and olfactory disgust; see Fig. 1). Compared to the control, all disgust-related sensory reminders significantly increased the probability that the individual remembered the prospective memory and acted accordingly (e.g., washed their hands after handling vegetables). The olfactory disgust stimuli showed the highest significance difference compared to the control (*p* < 0.001) while visual disgust and auditory disgust showed smaller responses, but they still had significantly higher probability to increase hand washing attempts than the control (for all cases *p* = 0.025). For example, participants were 14 times more likely to wash their hands when the disgust olfactory cue was presented compared to when the control was presented. Additionally, the disgust olfactory cue had a significantly larger effect than the auditory or visual cues (*p* = 0.001), and there was no difference between the visual and auditory disgust treatments (*p* = 1.00).

Among hand washing attempts taken by participants, there was no difference of hand washing performance among treatments [*F*(3, 64) = 0.72, *p* = 0.55]. Additionally, evaluating the ongoing prospective memory tasks (rearranging objects), there was no difference in the average time taken to complete the tasks among the conditions [*F*(3, 1146) = 2.54, *p* = 0.055]. However, there was a significant difference in correctness rate of the completed tasks among the four conditions (*χ*^2^ = 30.53, *p* < 0.001). Here, the probability of completing the tasks correctly was significantly higher in the visual disgust condition than all other conditions (*p* < 0.001) with the auditory disgust condition having the smallest correctness rate.

## Discussion

A significant finding in our study is how control-like posters, typical of hand washing interventions, may be ineffective at maintaining desirable behaviors such as hand hygiene. A cause for this may be miscommunication among individual of diverse cultures or these stimuli may simply be overlooked^13^,^29^,^30^. According to Po, Bourquin, Occena, & Po (2011), due to the diverse workforce in the modern food service industry, visuals or text prompts that are used in food safety interventions must be cross cultural and multilingual in order to be effective. Similarly, Nieto-Montenegro *et al.* (2008) prescreened their motivational materials and visual stimuli for cultural understanding, and made the appropriate modifications before implementing their intervention to increase hygiene practices with Hispanic workers in the mushroom industry. This intervention, based on Health Action Model (HAM), significantly increased hand washing.

Secondly, typical visual prompts may have been overlooked or not effectively engaging for the target audience. For instance, Guynn, McDaniel & Einstein (1998)^31^, in “a paper-based word association task”, showed no differences in the proportion for participants completing the PM task when given a basic reminder (e.g., “*Remember the three words that you studied at the beginning of the experiment*.”) and no reminder, and later found increases in PM task completion when target and action reminders were used in combination. In that study, the authors concluded that prospective remembering occurs because an associative link is activated past some threshold such that presentation of the target event automatically elicits the representation of the intended activity; however only reminders that incorporate the target intention plus another component are effective. Reversely, our visual control (e.g., CDC poster) incorporated an action without the target and the lack of effectiveness (compared to only using the target) could be similar.

Relative to the visual control, disgust cues effectively increased the probability of individuals remembering to act on a planned behavior. These results support the idea that distinctive, novel cues can be more effective at initiating planned behavior^32^, and our findings are in line with research that has shown increases in hand hygiene activity with disgusting visuals^17^,^19^. For instance, Porzig-Drummond *et al.* (2009), in a two part study, showed that priming an individual with disgusting videos (e.g., someone sneezing with residual snot) can effectively increase the initiation of hand washing with objects that are not visible dirty. They subsequently placed disgust/education-based posters in two bathrooms and educational posters in two other bathrooms, exhorting participants to wash their hands, and found that the disgust-based intervention was significantly better at promoting hand hygiene.

In our study, we show this stimulus effect to be cross-cultural by increasing the awareness of the intended hand washing behavior among Hispanic/Latino populations. Similarly, a national hand washing program in Ghana used disgust to motivate hand washing after changing a diaper or going to the bathroom thus increasing self-reported hand washing using both soap and water before eating by 30%^33^. To this degree, disgust, as shown in other studies, is a universal emotion that drives the behavioral avoidance of infectious disease and can be leveraged to increase hand washing among different ethnic groups^34^.

Additionally, the disgusting odor (“rotten fish”) proved to be the most effect prospective memory reminder. Prospective memory research has shown that salient or unusual stimuli, may produce involuntary orienting responses that are neither executive nor self-initiated direction thus reducing the resources needed for retrieval of the PM task^35^. This may explain part of the odor induced effect since the ongoing tasks under the odor condition did not have additional costs (e.g. average time to complete tasks) compared to the other conditions. However, to quickly discount this effect as an attentional response to a salient reminder would be unjustified since the disgusting sound in another condition showed a significantly smaller effect. Another type of automatic process in prospective memory is memory-based. To this account, odor has long been associated with memory, and more importantly, this odor-memory association is highly correlated with emotion^26^,^36^. For instance, Hertz and Schooler (2002) showed that autobiographical memories induced by odor was experienced as more emotional, and associated with stronger feelings of being brought back in time to the initiation of the event compared to memories evoked by verbal or visual cues. Additionally, odor stimulus has been shown to produce larger, more startled emotional responses than visual or auditory presentations^37^,^38^. Our work supports these findings and further supports recent studies that this odor-memory association in context with a disgusting emotion can help engage individuals to act on an intention of self-protection thus providing a unique tool for behavioral interventions^21^. Here, the odor of disgust evoked avoidance, a common signal from the olfactory system which warns about microbial threats^22^,^23^,^28^, and this avoidance reminded an individual to perform the intended decontamination PM target of washing their hands.

To date, most prospective memory (PM) studies have been limited to paper-based word associations, computer-based or board game simulations which are unable to test more complex human behaviors that involve both cognitive and physical engagements^31^,^39^. For example, Rendell & Craik (2000) showed age-related differences in time and event-based PM tasks using Virtual Week, a popular board game that stimulates real-life PM actions without actually performing them. Similarly, more naturalistic event-based studies conducted outside the lab can only look at simple tasks and do not benefit from the controls offered from more traditional laboratory studies^40^. Our study provides a laboratory design to study complex behaviors associated with memory and measures differences in environmental changes that influence these behaviors. For instance, this study observed the PM task of engaging in a hand washing behavior under different ambient conditions while maintaining laboratory control. Additionally, incorporating realistic situations into the design allows measurements of the actual application and allows practitioners to easily implement study findings.

## Methods

This study was conducted according to the Declaration of Helsinki for studies on human subjects. The protocol was approved by the Institutional Review Board of the University of Arkansas (Fayetteville, AR). The experimental procedure was explained to participants and a written informed consent was obtained from each prior to participation.

### Experiment 1

This preliminary experiment was performed to create a statistical model for determining hand washing performance by recording the variance of the measurements taken by the HHV that will later be used in Experiment 2.

### Physical surrogate

GGP (Glo Germ™, Inc., Moab, UT) was chosen as a physical surrogate for a food borne pathogen contamination. GGP is a non-toxic agent which has been previously shown to simulate *L. monocytogenes* cross-contamination in a food service environment, due to its small particle size (5 μm, compared to 1–2 μm for *L. monocytogenes*). Additionally, GGP fluoresces under ultraviolet lighting which permits quantification of very low concentrations perhaps as low as 1/10 the levels of a non-fluorescent surrogate and the ability to rapidly quantify amounts transferred among surfaces^41^.

A 1:10 w/v suspension of the physical surrogate Glo Germ™ polymer powder (GGP) was prepared by adding 1 g of GGP to 10 ml of 70% ethanol, followed by vortexing for 1 min.

### Electric Hand Hygiene Verification (HHV) Machine

To measure variables related to hand hygiene performance, an electronic hand hygiene (HHV) machine constructed in conjunction with researchers at Oklahoma State University was setup in our lab^42^. Using the HHV, allowed assess real-time metrics for analysis of several nominal and continuous variables associated with the handwashing process according to the World Health Organization (WHO) hand-hygiene guidelines (see Table 1 for technical description). All recording components was monitored by the main microcontroller (Micro-programmed Control Unit, MCU; v1.22, Seeeduino Mega, Seed Studio, Shenzhen, China), and Wi-Fi modules were put into both the automated paper dispenser and automated soap dispenser (Wifly GSX Wireless Module, Microchip Technology Inc., Chandler, AZ) to minimize visible cords. The hands motion detection unit (Microsoft Kinect Sensor, Microsoft Inc, Redmond, WA) monitored the movements of hands in a 3D space and measured the real-time hand washing actions (hand washing and lathering duration) of a human participant.

### Procedure

Six volunteers (3 men and 3 women), ages ranging from 23 and 43 years (mean age ± standard deviation = 29 ± 8 years) participated in the preliminary study. First, both hands of the participants were scrubbed clean with an alcoholic wet wipe and allowed to air dry. Afterwards, 300 μl of the concentrated GGP suspension was spread evenly over the surface of each hand and participants were asked to rotationally rub their hands in a figure eight pattern (covering both the front and back of their hands) until the solution dried. Participants were then asked to wash their hands for a specified period of time with different conditions where their hand washing variables were tracked using the HHV. Three times (5 seconds, 10 seconds, 15 seconds) and four conditions (no soap + no paper towel, paper towel + no soap, soap + no paper towel, soap + paper towel) were used in this initial study. In total, the six participants completed 12 washing trials with each condition being partitioned randomly across three separate days. Participants were not informed of other variables being recorded by the HHV.

**Figure 2:**
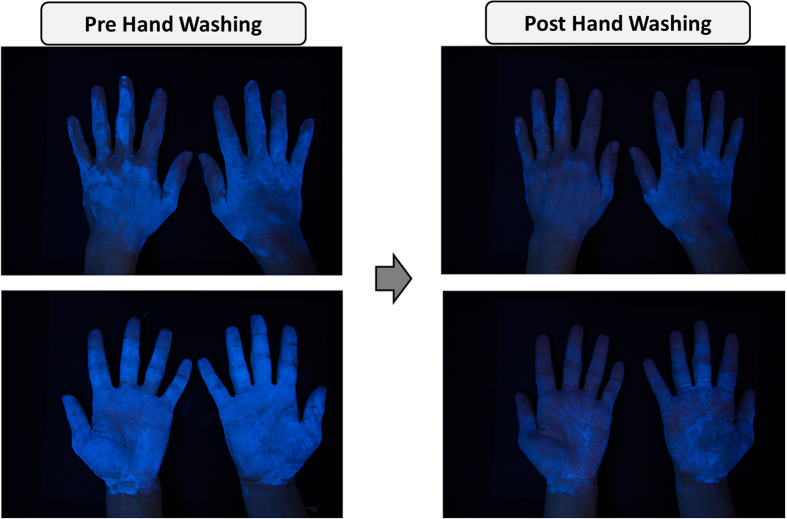
Hand contaminated with GermGlo pre and post hand washing. The picture on the screen was taken at the Food Science department building, Copyright Bob Pellegrino of University of Arkansas.

A baseline picture of both the front and back of the participants’ hands were taken prior to any of the trials. Additionally, the front and back of both hands of the participant were photographed pre and post hand washing trial to measure changes in GGP concentrations. Photographs were taken in a darkened room using a digital SLR camera positioned 31 cm above the hand, and set to manual settings (Canon EOS 5D Mark II Full Frame DSLR Camera, EF 24–105mm f/4 L IS USM Lens, focal length = 35 mm, shutter speed = 1/30 s, aperture = f-stop 5.6, ISO = 400). The only light source were 4 ultraviolent spiral-shaped bulbs (PLT Inc., 13W bulb) on each corner (at a 20 cm distance and 30º degree angle from the hands) of a table top camera stand with a parallel tripod (holding the camera). Camera setting and positioning were selected based from a previous study quantifying GGP^41^. For an example of a pre and post washing trial picture of hands with this procedure, see Fig. 2.

### Processing Images to Determine Amount of GGP on Hands

Image processing tools in MATLAB^®^ (ver. 8.3.0532, The MathWorks R2014a, Natick, MA) were used to quantify the amount of GGP (ppm) on each hand (including baseline reflectance). The amount of GGP transfer was quantified by an algorithm that determined specific thresholds for each hand (changing the image to binary) to filter out background noise, followed by multiplying the binary image pixel values by the original image pixel values and summing the pixel intensities in the sample area^43^. To calculate total hand illumination, the base line binary green component (of the back and front of the hands) were subtracted from the pre and post hand washing binary green component (of the back and front of the hands) and the difference between corrected pre and post hand washing components were recorded.

**Figure 3:**
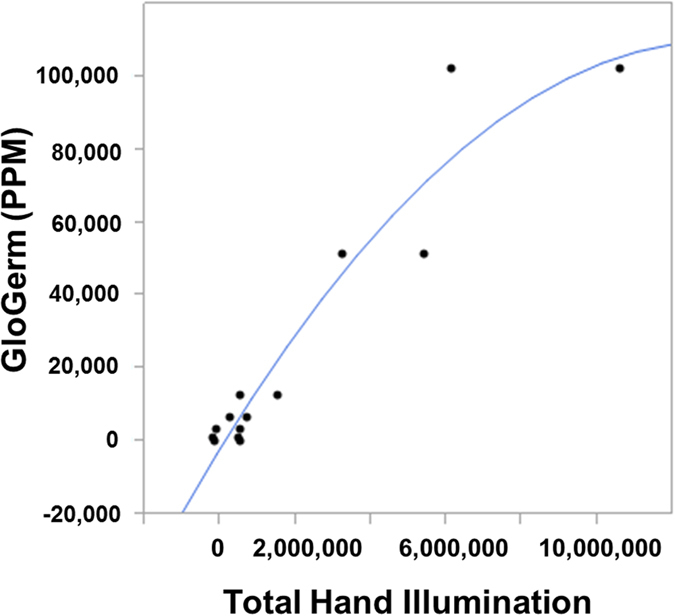
Quadratic calibration curve for total hand illumination *vs.* concentration of GGP (*N* = 2).

The total hand illumination was related to the concentration of GGP by creating a calibration curve for a two sets of hands (one male and one female; mean age = 23 years). This was done by spreading a known serially diluted GGP concentrations ranging from 200 to 102,400 ppm of GGP on the front and back of a volunteer hand. The computed intensities were plotted against concentration, and a second-order calibration equation was chosen from observations and the application of the equation (see Fig. 3). This equation was used to define the performance of hand washing in the model described below.

No observations had a Cook’s D influence^44^ larger than 1 thus no outliers were excluded from the analysis (*N* = 72). Looking at the correlations between GGP concentration (based on the equation mentioned above) and the continuous variables recorded by the machine [lathering time (seconds), water temperature (Celsius), water usage (liters), hand washing time (seconds), and paper towel weight (grams)] there were no correlations for water usage (*r* = –0.16), temperature (*r* = –0.009), and paper weight (*r* = 0.05) and thus were removed from the model. Next, a mixed model with GGP concentration (based on the equation mentioned above) was set as the dependent variable and the remaining continuous variables with recorded by the machine and their interaction were set as its predictors, and individual participants as a random predictor. Furthermore, data was normally distributed and residuals were checked for appropriateness, including any issues with multicollinearity among predictors (VIF < 2).

### Experiment 2

Using a real-time prospective memory scenario, Experiment 2 compared hand washing behaviors and performance across sensory reminders and a control with the HHV machine model developed in Experiment 1.

### Participants

Eighty-three volunteers (26 men and 57 women) with an age range age range from 18 and 44 years (mean age ± standard deviation = 25 ± 5 years) participated in the study. All participants were of Hispanic/Latino descent and reported that they had no history of major diseases, and no sensory or cognitive impairments. Additionally, a prescreening survey was used to exclude individuals with unusual sensitivity disorders such as obsessive-compulsive disorder towards washing and to measure individual differences in odor perceptions. This survey included an 11-item washing subscale of the Maudsley obsessive–compulsive inventory (MOCI) to assess each participant’s level of fear of contamination^45^ and determined perceived valence among 6 common odors (3 pleasant and 3 unpleasant) by asking surveyors for their degree of agreement with the statement (“*When I smell the following odor I feel unpleasant/disgusted.*”) on a 7-point Likert scale ranging from 1 (“strongly disagree”) to 7 (“strongly agree”). This survey also asked openly to “please describe what language(s) were primarily spoken in your childhood home.” Participants were informed that the study concerned emotions and behavior, but no further details were provided.

### Sensory Cues

Three disgust-related sensory cues representing sight, hearing and smell and one visual control were used as prospective memory reminders. The visual and auditory cues were selected from the International Affective Picture System (IAPS) and the International Affective Digital Sounds (IADS), respectively, and the emotional dominance of disgust (among male and female) measured in two separate studies that determined discrete emotions of both databases on a 7-point and 9-point scale^46^,^47^,^48^,^49^,^50^. For example, the visual cue chosen (IAPS 9300) was a picture of dirty, overused toilet having means of 2.26 (±1.76), 6.00 (±2.41), and 4.12 (±2.57) for valence, arousal, and dominance and a high disgust mean (6.00 ± 1.19), while the audio cue chosen (IADS 702) was an auditory belch having means of 4.45 (±2.57), 5.37 (±1.95), and 5.23 (±2.04) for valence, arousal, and dominance and a high disgust mean (7.38 ± 1.91). The [odor trimethylamine (“rotten fish”)] was chosen from the results of a pre-screener filled out by the participants that measured terms of unpleasant/disgust and pleasant/happiness appropriate for the Northwest Arkansas region^51^. For the control, a conventional hand-hygiene reminder poster approved by the Center of Disease Control (CDC) was used^52^.

### Procedure

Prospective memory refers to the act of planning an act in the future. This mechanism is the opposite of retrospective memory which refers to remembering information learned in the past^53^. Similar to other prospective memory tasks, a cover task (or ongoing task) was used in conjunction with prospective memory events^31^,^54^. For this study, an event-based prospective memory procedure was used with one intention. This intention (or event) was for the participants to wash their hands after any activity that involved touching vegetables, rocks, cotton, or metal. This prospective memory event and triggering items were read out loud to subject off a handout which they were allowed to study for up to one minute. Additionally, items listed on this handout were randomized per participant, and directions and words were in English (top of page) and the participants’ childhood language (bottom of page).

Next, participants were given two distraction tasks which were used as a buffer between prospective memory instructions and the ongoing tasks instructions. For the first distraction task, participants were asked to complete seven 6th grade level math problems in five minutes. The second distraction task was a retrospective task where participants were shown 30 random words (displayed on a screen one at a time with a 2 second duration per word) and after their presentation were given two minutes to recall (write down) as many words as they can remember.

After the distraction tasks, participants were read and given a piece of paper informing them that in the next test, they will be asked to perform the following three ongoing tasks to a series bins full of objects of varying sizes.

- Task 1: Arrange the items in a row from big to small.
- Task 2: Arrange the items in a row alternating the biggest and the smallest.
- Task 3: Arrange the items in a row from small to big.

They were instructed to memorize these tasks by their number for up to one to two minutes. The directions and tasks of this handout were in English (top of page) and the participants’ childhood language (bottom of page). Participants were then escorted into the testing facility to begin the testing procedure without the benefit of being able to refer back to the task directions. In the test facility, four bins labeled A, B, C or D were positioned in alphabetical order on a long table. Each bin contained one of the following object sets of varying sizes: 12 fresh tomatoes, 12 colorless sticks, 12 colorless balloons and 12 paper rings. These object sets were randomly assigned to bins per test trial. Next, the test administer called out a number and a letter, representing the task for the participant to perform on a particular bin of objects. Participants were asked to perform a randomized set of 15 tasks on the bins; however, only 2 (or 20%) of these tasks involved handling the bin of tomatoes.

Throughout the ongoing tasks, participants were subjected to one of three cues or a control. The control/visual cue and odor were constantly present via poster (positioned at eye level in the middle of the bins) and a hidden vaporizer respectively. Furthermore, the vaporizer had 140 mL of water with 500 μL of the trimethylamine-oil solution (50:1 oil to trimethylamine vortexed for 30 seconds). The audio cue played every 15 seconds with a recording time of 5 seconds at 70 dB SPL via speakers.

All tasks were performed in the University of Arkansas’ pilot test kitchen (Fayetteville, AR). In this kitchen, the HHV system was attached to a hand washing station installed next to an elongated table (where the main test took place) and included a sink, automated soap and paper towel dispenser. Additionally, a desk positioned cattycorner to the sink was used by the test administrator to instruct participants and monitor their results. Each hand washing event during a food handling task and its associated variables were recorded by the HHV, and the length and correctness of each task was recorded by the administrator. Furthermore, no immediate feedback was given to the participants regarding errors or other aspects of their performance.

### Analytical Procedure

Participants with a MOCI score seven or higher or reporting a fear of contamination and those under the odor condition not reporting an agreement that “rotting fish” was disgusting/unpleasant in the prescreening survey were excluded from further data analysis. This exclusion removed three participants (2 men and 1 woman), resulting in eighty remaining participants (24 men and 56 women), with ages ranging from 18 to 44 years (mean age ± standard deviation = 25 ± 5 years). These participants were further balanced across all four conditions by mean age [*F*(3, 78) = 1.05, *p* = 0.37] and gender ratio (for all conditions, 6 men and 14 women). Additionally, there was no significant difference of MOCI scores [*F*(3, 78) = 0.36, *p* = 0.78] or PM event study times [*F*(3, 65) = 0.88, *p* = 0.45] across treatments.

To determine the treatments that aided in memory planning and influenced hand washing behaviors a simple binary logistic regression model was used. The response was if the individuals washed their hands (yes or no) while the fixed predictor was the treatment in which they performed the tasks (control, visual disgust, auditory disgust, and olfactory disgust). Additionally, odds ratio tests were performed to measure difference between the treatments.

To examine the difference of hand washing performance across treatments a one-way ANOVA was performed between the predictive performance score means and the four treatments. The predictive performance score was calculated for each hand washing event by inputting the variables recorded from the electronic hand washing machine (lathering time, hand washing time, and water usage) into the performance model developed in Experiment 1. A statistically significant difference for all tests was defined as *p* < 0.05.

## Additional Information

**How to cite this article**: Pellegrino, R. *et al.* Using Olfaction and Unpleasant Reminders to Reduce the Intention-behavior Gap in Hand Washing. *Sci. Rep.* **6**, 18890; doi: 10.1038/srep18890 (2016).

## Figures and Tables

**Table 1:** Variables monitored and recorded by the electronic hand washing machine.

Parameter (unit)	Definition	Calculation Criteria
Soap Usage (drops)	The drops of soap used for current hand washing event	The microcontroller records the total number of soap drops.
Lathering Time (second)	Soap lathering time	Once system software detects the soap dispenser being activated, it will start a timer for this parameter. This timer adds an average frame time (1second/frame rate) for every processed frame, if only one hand is detected (lathering) and the system software detects hand under water running faucet over one second, the lathering timer will be stopped.
Paper Towel Usage (piece, gram)	The number and weight of the used paper towel (s).	The microcontroller on the Wi-Fi module inside the paper dispenser counts the total number of paper used through trigger signals from the motor inside the dispenser. A scale is placed under the waste receptacle measuring the weight of a used paper towel.
Water Temperature (°C)	Water temperature	The MCU reads the temperature sensor once per iteration and stores the reading in to a 128 elements temperature buffer. If water is being turned on, MCU will send an averaged buffer temperature reading once every two seconds. The server program monitors the serial communication data from MCU for message that contains “TEMP”, and extracts the water temperature data and puts it in to another buffer. One averaged temperature data from this buffer is recorded into the log file for one hand washing event.
Water Usage (liter)	The volume of water used during hand washing event	The MCU reads Hall Effect flow sensors (in both hot and cold water pipe lines) and calculates of water usage at the end of a hand washing event.
Hand Washing Duration (second)	Hand washing time including wetting time and rinsing time	The system software monitors the hand location. Once it detects the hand under a water running faucet, it will start a timer for this parameter.
